# Editorial: Emotional resilience for wellbeing and employability: the role of learning and training, volume II

**DOI:** 10.3389/fpsyg.2025.1670866

**Published:** 2025-09-09

**Authors:** Rasa Smaliukienė, Svajone Bekesiene, Sarka Hoskova-Mayerova

**Affiliations:** ^1^General Jonas Zemaitis Military Academy of Lithuania, Vilnius, Lithuania; ^2^Vilnius Gediminas Technical University - VilniusTech, Vilnius, Lithuania; ^3^University of Defence, Brno, Czechia

**Keywords:** emotional resilience, wellbeing, employability, learning, training, research methods

## 1 Introduction

Emotional resilience has become a significant topic for researchers and educators who seek to understand what helps young people cope with stress when changing jobs or entering the job market. While considerable attention has been devoted to resilience in early childhood and clinical settings, its role in relation to the transition to adult life, employability and the nature of work has received more attention only in recent years (see Vol. 1 of Emotional Resilience for Wellbeing and Employability: The Role of Learning and Training). Resilience is now viewed as both an individual trait and a competence that can be developed through educational interventions and workplace practices (Smaliukiene et al.).

The ability to adapt positively in the face of adversity is particularly important in work and career contexts, where emotional demands, uncertainty and changing conditions are constant. During the pandemic, studies documented the psychological strain experienced by students, employees, and job seekers, highlighting the ways in which stress, anxiety, and social isolation interfered with learning, motivation, and career planning (Dost, 2025). In this context, interest in emotional resilience as a potential enabler of employability and a buffer against negative outcomes has grown.

At the same time, scholars have highlighted the trainable nature of resilience, suggesting that structured interventions, educational practices and coaching models could develop these capacities. However, the evidence base on how emotional resilience can be developed through learning and training remains fragmented. Although progress has been made in identifying key emotional and cognitive factors, such as self-regulation and adaptability, there are still gaps in understanding of the pedagogical processes and conditions through which emotional resilience contributes to wellbeing and employability across different groups of people.

This Research Topic brings together ten studies that address these concerns. The issue includes empirical and conceptual contributions that examine the development of emotional resilience through training, coaching, leadership and institutional learning contexts. Taken together, the papers offer evidence-based insights into how emotional resilience is cultivated, how it supports professional development and the role that educational and organizational systems can play in enabling these outcomes.

## 2 First field: developing emotional resilience for employability through interactions between psychological, organizational, educational and physical training

Research shows that emotional resilience can be developed. It is also becoming increasingly important to understand the psychological and educational factors that help people to develop this skill. Emotional resilience does not depend solely on personality. It is also shaped by leadership style; the level of support people feel they receive from their organization and how well their personal values align with the work environment. The following studies explore how personal characteristics, and the work environment influence emotional involvement and resilience for employability.

### 2.1 Leadership and resource-driven engagement

Tang et al. focus on the role of coaching leadership in enhancing employee engagement in vocational and professional environments. In an empirical study involving 402 Master of Business Administration (MBA) and Executive MBA (EMBA) students, the researchers found that coaching leadership significantly increased engagement, measured through vigor, dedication, and absorption. These effects were partially mediated by organizational self-esteem and further moderated by individuals' learning goal orientation. The findings point to a model in which leadership does not only direct but allows, creating conditions for employees to assume value, confidence, and purpose (Tang et al.). This study makes an important contribution to resilience research by strengthening the idea that positive emotional conditions such as enthusiasm and energy that are not isolated experiences but emerge in interaction with supportive social contexts. The implication is that engagement-enhancing leadership could be a developmental pathway for increasing emotional resilience, particularly in roles that demand adaptability and sustained motivation.

### 2.2 Training in psychological safety and secured leadership

Based on organizational perspective, Navas-Jiménez et al. explore emotional resilience in the military context through the lens of secure base leadership (SBL). drawing on attachment theory and the job demands–resources model, their study with 363 military cadets reveals that SBL increases emotional resilience indirectly through its impact on work (service) engagement. Here, leadership provides not only direction but also emotional reassurance, autonomy support, and psychological safety, which are conditions that help trainees internalize challenges as developmental opportunities rather than stressors. They also found that the mediating role of engagement is very important: the study suggests that resilience is not rooted directly, but fostered through motivational mechanisms tied to perceptions of safety, relatedness, and challenge. These findings are especially relevant for high-stress environments, where leadership must simultaneously increase discipline and emotional support. The results underscore the developmental potential of leadership models that balance authority with trust and individualized guidance.

### 2.3 Training and developing employability in vocational education

Li et al. examine how collaboration between universities and industry enhances vocational students' perceived employability. This addresses a gap in research on the role of macro-level institutional factors in vocational education, an area which is often underrepresented in employability discourse. Using data collected from 341 vocational college students, the study reveals that a culture of quality, defined as a collective institutional dedication to enhancement, ethical principles, and performance, significantly enhances students' perceptions of their career preparedness. The authors recommend integrating industry-oriented learning into curricula and reinforcing internal systems that foster a culture of excellence. They conclude that employability is not merely the outcome of skill development, but rather the result of a coherent institutional strategy, effective stakeholder engagement and active student participation in educational processes.

### 2.4 Physical fitness training for greater resilience

Physical fitness is a cornerstone of military readiness, directly contributing to operational effectiveness and reducing the risk of injury. Within this framework, aerobic endurance is of particular importance. Using data from 486 military recruits and cadets, Drozd et al. address a knowledge gap in the application of ergometer-based metrics to predict field endurance outcomes. The results suggest that the current model may misrepresent individuals' physical readiness, which has implications for the accuracy of recruitment and the adaptation of training. More broadly, the findings emphasize that personalized endurance training enhances physical capability and fosters psychological resilience, preparing personnel for the physical and mental challenges of military service. Therefore, updated assessment protocols and training designs need to incorporate physiological training in order to develop resilience for performance-oriented military service.

Taken together, these studies emphasize that emotional resilience for employability is not developed through efforts made in isolation within the study curriculum, but rather through the interaction of psychological, organizational, educational and physical systems. In professional, educational or military environments, resilience develops when individuals are encouraged to engage meaningfully with challenges through leadership that fosters trust, institutions that align learning with opportunities, and training that builds mental and physical resilience. These findings highlight the importance of designing study curricula that integrate not only skill-focused, but also structurally embedded, resilience-building initiatives.

## 3 Second field: coaching and support for employability and career development

Coaching and support are crucial in helping individuals adapt to the demands of modern labor markets, especially those in transitional or vulnerable professional situations. The six contributions in this Research Topic emphasize that employability and emotional resilience are developed through a combination of personal attributes and structured interventions, institutional environments and socio-cognitive mechanisms.

### 3.1 Coaching and support for employability and career development

Coaching has emerged as a vital means of fostering individual resilience and promoting long-term employability. Focusing on this intersection, Sipondo and Terblanche conducted a review of 51 studies to determine the extent to which organizational coaching contributes to workplace resilience. They identify a clear knowledge gap: despite the growing popularity of coaching as a developmental intervention, the existing body of research is fragmented and there is limited empirical evidence on the impact of coaching on resilience-related outcomes such as adaptability, self-efficacy and career engagement. They call for evidence-based coaching models to be developed that align coaching goals with resilience-building processes. They also recommend further research into the mechanisms that link coaching to employability and resilience outcomes.

### 3.2 Career development and employability among unemployed adults

Carvalho et al. evaluated the effectiveness of a career intervention based on Social Cognitive Career Theory (SCCT), which was proposed by Lent and Brown (2013). The aim was to enhance employability resources among unemployed adults. The study used a quasi-experimental longitudinal design to follow two intervention groups (one face-to-face and one online) and a control group. Notably, the researchers identified distinct patterns of influence for different employability dimensions. For instance, career identity and self-management resources had the greatest impact in the short term, whereas human capital and professional development became more significant over time. Meanwhile, social capital and networking showed the greatest influence immediately after the intervention. These findings suggest that the different facets of employability may respond differently to intervention stimuli depending on the timing and context of exposure.

### 3.3 Institutional culture for learning and employability in vocational education

Li et al. investigated the influence of institutional quality culture on students' perceived employability. The study introduces a conceptually rich definition of quality culture, integrating structural dimensions (e.g., accountability systems) and cultural dimensions (e.g., shared values and continuous improvement). Drawing on Huang's educational philosophy, the researchers argue that institutions of education and training must do more than simply teach skills; they must also foster professional ethics, collaborative habits and a sense of broader purpose in their students, thereby aligning education with the needs of the labor market and society. Empirically, the results demonstrate that students who perceive their school as being committed to providing high-quality teaching, ethical development and continuous improvement are more confident in their own employability. The study offers two significant contributions. Firstly, it provides a localized, culturally anchored model of how employability is developed in Chinese vocational education. Secondly, it empirically validates the theoretical claim that a quality culture alone is insufficient unless it is paired with industry linkages that convert educational efforts into experience that is relevant to the labor market.

### 3.4 The role of organizational support

While leadership and self-esteem are critical resources, individual differences in personality also contribute to resilience-related outcomes. Sun et al. investigate the relationship between conscientiousness and work engagement, focusing on the mediating effect of presenteeism and the moderating effect of perceived organizational support (POS). Using data from 376 employees, they demonstrate that conscientiousness predicts greater engagement but also increases the risk of presenteeism—continuing to work while unwell—which in turn negatively affects engagement. Notably, high levels of POS buffer this relationship, strengthening the positive impact of conscientiousness on engagement. This study adds a nuanced perspective to the research on emotional resilience by emphasizing the importance of conscientiousness. While conscientiousness is generally considered a positive trait, it can have negative consequences in unsupportive environments. Therefore, emotional resilience training must not only develop personal attributes, but also include organizational strategies that provide support and legitimacy in times of stress or illness. These findings confirm that personal and contextual resources are interdependent in fostering engagement and psychological functioning.

### 3.5 Support during workplace automation

Alshamsi et al. conducted a cross-sectional study to examine the relationship between the Technology Acceptance Model (TAM) and pharmacists' experiences of burnout and depression within the context of pharmacy robotic dispensing systems in the United Arab Emirates. Notably, the study found a negative correlation between TAM dimensions and mental health outcomes, indicating that pharmacists who found the technology more useful and easier to use experienced lower levels of burnout and depression. These results suggest that while automation in healthcare may not inherently reduce psychological strain, its effectiveness is closely tied to how well staff perceive and adapt to it. Furthermore, the findings imply that gendered experiences with technology and organizational demands may play a critical role in moderating the relationship between innovation and employee wellbeing.

### 3.6 Mediation for emotional resilience, grit, and gendered pathways to life satisfaction

Jia investigates how emotional resilience influences life satisfaction among teachers of Chinese as a Foreign Language (CFL), emphasizing the mediating roles of grit and employability, and the moderating role of gender. Drawing on data from 1,003 teachers in China, the study applies a chain mediation model and finds that emotional resilience has no significant direct effect on life satisfaction. Instead, its influence is fully mediated by grit and employability—two constructs critical for sustained engagement in demanding educational roles. Importantly, gender differences emerged in the structure of these pathways. This finding highlights the differentiated challenges and professional strategies experienced by male and female CFL teachers, and suggests that gender-responsive research may be needed to support teacher wellbeing across diverse cultural and institutional settings. The study is theoretically grounded in positive psychology and career adaptability frameworks, and contributes to research on emotional health in teaching by emphasizing that resilience is not only a personal asset but also a socialized and structurally shaped capacity.

Together, these studies emphasize that coaching and support for employability and career development should be considered multi-level processes shaped by personal, organizational and institutional dynamics. At the same time, career interventions, especially those targeting unemployed or at-risk populations, demonstrate that employability is developed in stages that reflect shifting psychological and social needs. Institutional environments, such as those in vocational education, further influence these outcomes by either reinforcing or undermining students' confidence in their career prospects. Finally, the interaction between personality traits and perceived support highlights the limitations of individual strengths when they are not reinforced by positive workplace or educational contexts. Overall, the findings suggest that meaningful progress in terms of employability and resilience requires a coordinated approach involving coaching models, the timing of interventions, institutional culture and organizational support.

## 4 Further perspectives

The studies in this Research Topic collectively support that emotional resilience is not a fixed personal trait, but rather a dynamic competence which is sensitive to context and can be developed through structured training, targeted support, and education and employment systems. This volume's key contribution is demonstrating that resilience and employability develop together through the interaction of personal attributes (such as self-regulation, career identity and conscientiousness) with organizational, institutional and technological resources.

Several areas for future research emerge. Firstly, the contributions of Research Topic highlight the need for integrative frameworks that exceed isolated constructs and embrace models capable of capturing the interaction of emotional, social, cognitive and physical resilience resources over time. For instance, studies by Carvalho et al. and Jia, among others, reveal distinct temporal patterns in the development of employability capacities. This suggests that longitudinal studies are essential for understanding when and how interventions have an effect.

Secondly, the findings emphasize the importance of adapting interventions to the characteristics of specific populations, such as unemployed adults, vocational students, educators, and military recruits. Emotional resilience is shaped by individual learning processes, institutional culture (Li et al.), leadership style (Navas-Jiménez et al.), and the perceived impact of technological change (Alshamsi et al.). These contextual variations demand a more granular approach to building resilience.

Thirdly, there is a need to better integrate the physical and psychological dimensions of resilience. Drozd et al. demonstrate that physical endurance training predicts not only operational readiness, but also prepares individuals psychologically for stress, challenges, and adaptation. Future research could examine how physical training interacts with emotional learning to enhance resilience and employability.

Finally, while this volume presents robust empirical findings, it also highlights methodological limitations. The most significant of these is the reliance on cross-sectional designs. There is a need for longitudinal, mixed-methods, and experimental designs that can more accurately trace the development of resilience.

Taken together, the articles in Research Topic point to a conceptual shift from viewing resilience as an individual psychological resource to recognizing it as a shared responsibility that is cultivated through pedagogical design, institutional commitment, and ongoing support throughout working life.
